# An Ultra-Brief Proxy Measure for Early Mental and Substance Use Disorders and Suicide Risk Case Detection at the Community and Household Level: An Efficient and Feasible Clinical and Population-level Service Needs Screening Tool

**DOI:** 10.18103/mra.v11i10.4381

**Published:** 2024-01-11

**Authors:** Melissa A Stockton, Ernesha Webb Mazinyo, Lungelwa Mlanjeni, Kwanda Nogemane, Nondumiso Ngcelwane, Annika C. Sweetland, Cale Basaraba, Charl Bezuidenhout, Griffin Sansbury, Kathryn L. Lovero, Maria Lídia Gouveia, Palmira Fortunato dos Santos, Paulino Feliciano, Wilza Fumo, Antonio Suleman, Maria A. Oquendo, Christoffel Grobler, Melanie M Wall, Phumza Nobatyi, Andrew Medina-Marino, Milton L. Wainberg

**Affiliations:** 1 Department of Epidemiology, Gillings School of Global Public Health, University of North Carolina at Chapel Hill, Chapel Hill, NC; 2 University of California Global Health Institute, University of California, San Francisco, USA; 3 Research Unit, Foundation for Professional Development, Buffalo City Metro, Eastern Cape Province, South Africa; 4 The Desmond Tutu HIV Centre, University of Cape Town, Cape Town, South Africa; 5 Eastern Cape Provincial Department of Health, Buffalo City Metro Health District, South Africa; 6 Department of Psychiatry, Columbia University Vagelos College of Physicians and Surgeons, New York, USA; 7 New York State Psychiatric Institute, New York, USA; 8 Department of Population and Family Health, Columbia University Mailman School of Public Health, New York, New York, USA; 9 Department of Biostatistics, Columbia University Mailman School of Public Health, New York, New York, USA; 10 Department of Global Health, Boston University School of Public Health, Boston, MA, USA; 11 University of North Carolina-Project, Malawi, Lilongwe, Malawi; 12 Department of Sociomedical Sciences, Columbia University Mailman School of Public Health, New York, USA; 13 Ministry of Health of Mozambique, Mental Health Department, Maputo, Mozambique; 14 Department of Psychiatry, Perelman School of Medicine, University of Pennsylvania, Philadelphia, PA, USA; 15 School of Health Systems and Public Health, Faculty of Health Sciences, University of Pretoria, Pretoria, South Africa

**Keywords:** Proxy Screening, Validation, Mental Health, South Africa, Community Care

## Abstract

Valid mental and substance use disorders and suicide risk screening tools are needed for community case finding of individuals who may not otherwise seek care. We evaluated the Proxy Mental Wellness Tool-3 (mwTool-3-proxy) a three-item screener that asks about the mental health of another adult, against a diagnostic gold standard in Mozambique and South Africa. The mwTool-3-proxy adapts the three items of the Mental Wellness Tool-3, developed in Mozambique using Mini International Neuropsychiatric Interview diagnoses as the criterion standard, regression modeling and expert consultation to determine the best three items for identifying any mental disorder. The Mental Wellness Tool-3 has been validated in South Africa, Spain and the United States, and is being validated in three countries in the Asia-Pacific and Israel. Pairs of adults in South Africa and Mozambique at primary and tertiary healthcare facilities were separately screened with the mwTool-3-proxy and diagnosed using the Mini International Neuropsychiatric Interview. We calculated the sensitivities and specificities for predicting any mental and/or substance use disorder and suicide risk among the proxy individual. We performed additional analyses restricted to respondents who were relatives of one another and who lived in the same household. The prevalence of any Mini International Neuropsychiatric Interview-diagnosed disorder among the 229 pairs in both countries was 35.6% (38.5% in Mozambique; 32.9% in South Africa). The pooled sensitivity of the mwTool-3-proxy for identifying any disorder among the proxy individual was 73.01 (95%CI: 65.5-79.65) – 70.24 (95%CI: 59.27-79.73) in Mozambique and 80.00 (95%CI 69.17-88.35) in South Africa. The mwTool-3-proxy is a culturally-relevant, ultra-brief valid measure that can improve mental and substance use disorders and suicide risk case detection with strong sensitivity at the community and household level and offer a means to efficiently and feasibly collect clinical and population-level service needs data.

## Introduction

In sub-Saharan Africa, there is a high burden of mental health and substance use disorders.^1,2^ For example, approximately 30.3% of South Africans will experience a mental disorder in their lifetime ^3^, yet only few received needed mental health treatment ^4^. Similarly, in Mozambique, a representative survey suggests a 20% prevalence of past-year depressive symptoms and 17% prevalence of lifetime suicidal ideation, yet, clinical mental healthcare is rarely sought ^5,6^. This is due in part to a lack of mental health specialists, underdeveloped community-based services, and low mental health literacy ^7–11^. Task-shifting mental health screening to the primary and community care settings has been identified as a promising strategy to increase access to mental healthcare in sub-Saharan Africa and other low-resource settings ^9,12,13^. Several task-shifting mental health treatment strategies are being implemented in multiple countries, including in sub-Saharan Africa ^13–16^. In order to scale up effective task-shifting treatment strategies, valid tools that have been translated into the local language are needed for non-specialists to identify individuals with mental health conditions at the clinic and community levels ^17^.

The Proxy Mental Wellness Tool-3 (mwTool-3-proxy) is an ultra-brief comprehensive mental health screener that was developed in Mozambique to facilitate community-based screening for early detection of mental disorders ^18^. The mwTool-3-proxy consists of the first three items of the mwTool-12. The mwTool-12 is a two-step 12-item screener that includes: 1) three initial questions to identify individuals with any probable mental disorder; followed by 2) nine additional questions to categorize individuals with a probable common mental disorder (CMD), severe mental disorder (SMD), alcohol use disorder (AUD), and/or suicide risk (SR) ^18^. The mwTool-12 was developed from a battery of 99 items from existing validated scales across nine commonly used mental health, substance use disorder, and global functioning assessments ^19–23^. Reduction from 99 items to 12 used the least absolute shrinkage and selection operator (LASSO) variable selection technique and expert clinical consultation ^18^.

The purpose of the mwTool-3-proxy is to allow clinic-based or community healthcare workers to ask a single individual three questions about the mental health of other adults – ideally household members or relatives – to help identify those in need of additional screening and potential intervention. While the mwTool-3-proxy has the potential to serve as an efficient and feasible clinical and population-level services needs screener, the tool has yet to be assessed outside of Mozambique ^18^. This study evaluates 1) the mwTool-3-proxy against a diagnostic gold standard, the Mini International Neuropsychiatric Interview (MINI) in Eastern Cape Province, South Africa and 2) pools data from the original validation study in Mozambique to compare performance across countries.

## Methods

The Mozambiquan data comes from the larger study developing and validating mwTool-3-proxy.^18^ The South African data was part of a larger study validating a battery of mental health screeners ^24,25^. This paper describes the performance of the mwTool-3-proxy in the two countries.

### STUDY SETTING

In Mozambique, data were collected from three primary care clinics in Nampula, Mozambique from December 5 to 12, 2018. In South Africa, data were collected at four government primary healthcare facilities and one provincial tertiary hospital all located in the Buffalo City Metro Health District, Eastern Cape Province, South Africa from February-May 2022.

### STUDY POPULATION

In Mozambique, adults (patients and accompaniers 18 years or older) able to communicate in Portuguese at the study facilities were eligible to participate in the larger parent study. In South Africa, any adult aged ≥18 years presenting at one of the study health facilities and able to communicate in isiXhosa or English were eligible to participate in the larger parent study. All participants responded to the MwTool-12 questions and the MINI about themselves. Those seeking care or accompanying another adult (regardless of their relationship to one another) were eligible to be enrolled as a dyad, respond to the Proxy mwTool-3 questions, and be included in this analysis.

### MEASURES

In Mozambique, the mwTool-3-proxy items had been translated into Portuguese using a robust process for forward and backward translation.^18^ The Mozambiquan study also used the Brazilian version of the MINI Plus.^26,27^ In South Africa, English versions of the mwTool-3-proxy and MINI were translated into isiXhosa using a the same robust process of forward and backward translation and review by the study staff and psychiatrist ^24^.

#### mwTool-3-proxy:

While the mwTool-3-proxy was developed out of the robust process used to develop the mwTool-12,^18,28^ the mwTool-3-proxy ultimately includes three items derived from the Patient Health Questionnaire-9 and General Anxiety Disorder-7 adapted to ask about the other adult.^20,29^ “In the last 2 weeks, how often did this person: 1) feel down depressed or hopeless; 2) feel nervous, anxious or on edge; and 3) been so restless that it’s hard for them to sit still.” All three questions are answered on a four-point likert scale including: “not at all;” “several days; “more than half the days;” and “nearly every day.” Reporting “several days” or more to any of the questions is considered an indication that the other person may have a mental disorder.

#### MINI:

Mental disorder diagnoses were made using the MINI, a structured diagnostic interview ^30^. A diagnosis of “any disorder” included at least one of the following MINI diagnoses: severe mental disorder (SMD - manic episode, hypomanic episode, psychotic disorder); common mental disorder (CMD - major depressive episode, posttraumatic stress disorder, general anxiety disorder); alcohol use disorder (AUD - alcohol abuse or dependence); substance use disorder (SUD - substance abuse or dependence; and suicide risk (SR - moderate to high suicide risk). The MINI-V modules were used to diagnose all disorders, save for PTSD and SR which were diagnosed with the MINI-plus for brevity. All diagnoses were for current disorders, except for SMD, which included *current* manic episode, *current* hypomanic episode, and *lifetime* psychotic disorder, because patients with a history of psychosis require referral to specialists.

### DATA COLLECTION

Research assistants (RAs) approached potential participants and any other adults accompanying them at the study facilities, informed them about the study, and invited them to participate. In a private area, the RAs then assessed interested individuals for eligibility and consented and enrolled eligible individuals. RAs then captured information about whether they lived together and their relationship to one another. RAs separately screened each participant with the mwTool-3-proxy Portuguese (Mozambique) or in either English or isiXhosa (South Africa). Following screening, nurses (blinded to the results of the mwTool-3-proxy) separately administered the MINI to each individual.

### ANALYSIS

We used descriptive statistics to characterize the study population and present the prevalence of the MINI diagnosed disorders. Sensitivities and specificities for predicting any disorder among the proxy individual were calculated. Stratified analyses were conducted using the following categories of respondents: 1) who lived in the same household, 2) were relatives of one another, 3) were not diagnosed with a SMD, and 4) responded in isiXhosa (only in South Africa).

### ETHICAL CONSIDERATIONS

Participants provided written informed consent. The Mozambiquan study was approved by the Eduardo Mondlane University Institutional Health Bioethics Council (CIBS FM and HCM/54/2017). The South African study was approved by the Foundation for Professional Development Research Ethics Committee (8/2021) and the Eastern Cape Department of Health Research Committee (EC_202110_015). Both studies were approved by New York State Psychiatric Institute Institutional Review Board (no. 7479, Mozambique and no. 8272, South Africa). Individuals with MINI diagnoses were either referred to Nampula’s provincial psychiatric hospital (Mozambique) or onsite psychiatric staff or linked to necessary services in line with the South African integrated chronic services manual (South Africa).^31^

## Results

### PARTICIPANT CHARACTERISTICS

A total of 458 participants (229 pairs) are included in this analysis. The pooled prevalence of MINI-diagnosed disorder was 35.6% (n=163). (Table 1)

In Mozambique, 109 pairs of proxy participants were enrolled (n=218). Sixty-seven percent (n=145) were women, 96.8% (n=211) were black, and 31.8% lived in the same household. With respect to the reported relationship to one another, 48% (n=105) were relatives, 35% (n=77) were friends, 3% (n=7) were neighbors, 13% (n=28) were colleagues or worked together, and one person did not specify. In Mozambique, the prevalence of MINI-diagnosed disorders was as follows: any disorder (38.5%; n=84); CMD 28.4% (n=62); AUD 6.4% (n=14); SUD 0.9% (n=2); SMD 16.5% (n=36); SR 7.8% (n=17).

In South Africa, we enrolled 120 pairs of proxy participants (n=240). Sixty-one percent (n= 146) of these participants were women, 97.5% (n=234) were black, 52.9% reported a monthly average household income of under R1000, and 67.5% (n=162) lived in the same household. With respect to their relationship to one another, 70% (n=168) were relatives, 23.3% (n=56) were friends, and 5.8% (n=14) were neighbors. We also enrolled one caregiver/receiver pair (n=2). The prevalence of MINI-diagnosed disorders in South Africa was as follows: any disorder 32.9% (n=79); CMD 16.3% (n=39); AUD 3.3% (n=8); SUD 1.7% (n=4); SMD 15.0% (n=36); SR 3.3% (n=8).

### PERFORMANCE OF THE MWTOOL-3-PROXY

In the pooled analysis and in both countries individually, the sensitivity of the mwTool-3-proxy to identify any disorder among the other adult was >70%. This performance held when restricted to relatives or those without a MINI confirmed SMD diagnosis, household members, or to those who responded in isiXhosa. (Table 2)

## Discussion

In this study, the mwTool-3-proxy yielded high sensitivity for identifying any disorder among accompanying adults in the pooled analysis, and in both Mozambique and South Africa.

The mwTool-3-proxy offers a means to greatly improve early mental health case detection at the community and household level. This is because the mwTool-3-proxy only requires a lay-health worker to ask one individual three questions about their household members to identify individuals with a probable mental disorder in need of further assessment. Proxy respondents have been used to facilitate assessment in clinical care when the individual is unable to self-report, either due to being unavailable, incapable of providing a response, unaware of their diagnosis, unlikely to seek care due to stigma, or under-aged (recognizing that screening children necessitates pediatric specific tools) ^18,32^. For mental health, in communities with low mental health literacy and/or high mental health-related stigma, a proxy tool may also improve early case detection at the household level among individuals who may otherwise not have formal contact with the health system. In this way, the mwTool-3-proxy presents a novel means of mental health screening, by allowing a lay-health worker to quickly assess an entire household.

Few proxy mental health measures have formally been developed for and tested in LMICs. While other research among people with disabilities suggests that proxy measures for emotional or psychological distress may be less reliable than objective measures ^33^, our results show that the mwTool-3-proxy had good sensitivity for identifying any mental disorder among all pairs. The strong sensitivity held when restricted to household members and family members, suggesting this tool would work well in community settings. Importantly, besides its use in the community, clinic-based proxy assessments may help identify at-risk family members of the individual seeking care in an efficient and effective way. Future health programing in Mozambique and South Africa could use tools such as the mwTool-3-proxy to identify those in need of further mental health screening, thus improving access to services at the community level. However, additional research is needed to investigate the acceptability and appropriateness of proxy mental health screening in different settings. In particular, mental health programming will need to be culturally sensitive and attentive to any potential negative consequences from reporting on the mental health of other household members without their express consent.

While the mwTool-3-proxy yielded high sensitivities, the specificities ranged from 32.1-55.4%. The mwTool-3-proxy screener is not designed to function as a diagnostic tool, but as a proxy screener. Thus, higher sensitivity is prioritized to ensure that all of those who may require further screening and potential intervention are identified. Use of the tool as a first step to approximate prevalence and incidence should be followed by second-tier or follow-up assessment of proxy individuals to confirm mental health distress or diagnosis. Thus, the mwTool-3-proxy may have substantial utility at the population level, enabling the identification of whole communities where further mental health screening for early detection and treatment intervention may be warranted as well as population-based prevention and promotion efforts. Further, because the mwTool-3-proxy adapts items from self-report screeners ^20,29^, it may be feasible to use this tool in large-scale population assessments.

### LIMITATIONS

There are some inherent limitations to this study. The study included a sub-sample of pairs from a targeted sample of adults present at health care facilities and the prevalence data reported in this manuscript should not be interpreted as generalizable or representative of either the Mozambican or South African adult population. In South Africa, we did not have a large enough sample of individuals who were screened in English to separately validate the screener in English. Due to the SMD diagnostic procedures in Mozambique, we were unable to restrict the analyses to those without a *current* SMD. Nevertheless, it does not appear that including or excluding those with SMD (*current* hypomanic or manic episode or *lifetime* psychotic episode) effected the performance of the tool.

## Conclusion

The mwTool-3-proxy is a valid, ultra-brief measure that is culturally-relevant and validated, and offers a means to greatly improve mental health early case detection at the community and household level in Mozambique and South Africa. As well, the mwTool-3-proxy screening can be delivered both using paper format or as a digitized tool, supporting the advancement of mental health technology in LMICs ^34,35^. To our knowledge, it is also the first to be translated into isiXhosa and be validated for use among isiXhosa speakers. Proxy measures such as the mwTool-3-proxy, provide an opportunity to better identify communities where additional mental health screening and services may be needed. Further, the mwTool-3-proxy could be used to expand access to services in primary- and community-care settings.

## Figures and Tables

**Table 1:** Proxy Participants (N=458)

Mean (SD) or n (%)	Total (N=458)	Mozambique (n=218)	South Africa (n=240)
Age (Range: 18-78)	34 (12.5)	30.6 (10.3)	37.1 (13.5)
Sex			
Male	167 (36.5)	73 (33.5)	94 (39.2)
Female	291 (63.5)	145 (66.5)	146 (60.8)
Household members*			
No	224 (49.3)	146 (68.2)	78 (32.5)
Yes	230 (50.7)	68 (31.8)	162 (67.5)
Relationship			
Parent	28 (6.1)	6 (2.8)	22 (9.2)
Partner/Spouse	65 (14.2)	37 (17)	28 (11.7)
Child	26 (5.7)	6 (2.8)	20 (8.3)
Relative	154 (33.6)	56 (25.7)	98 (40.8)
Friend	133 (29)	77 (35.3)	56 (23.3)
Other	52 (11.4)	36 (16.5)	16 (6.7)
MINI Diagnoses			
Any Disorder	163 (35.6)	84 (38.5)	79 (32.9)
CMD	101 (22.1)	62 (28.4)	39 (16.3)
AUD	22 (4.8)	14 (6.4)	8 (3.3)
SUD	6 (1.3)	2 (0.9)	4 (1.7)
SMD	72 (15.7)	36 (16.5)	36 (15.0)
SR	25 (5.5)	17 (7.8)	8 (3.3)

CMD=Common Mental Disorder; AUD=Alcohol Use Disorder; SUD=Substance Use Disorder; SMD=Severe Mental Disorder; SR=Suicide Risk;

* 2 pairs (n=4 individuals) from Mozambique reported discordant information about living together and are treated as missing.

**Table 2:** Performance of the mwTool-3-proxy in Mozambique and South Africa for identifying any disorder among the proxy, restricted by sub-population

Population	# Cases (Prevalence)	# Screen Positive	Sensitivity (95% CI)	Specificity (95%CI)
**Total (N=458)**	163 (35.6%)	278	73.01 (65.5-79.65)	46.10 (40.31-51.97)
Household members* (n=230)	86 (37.4%)	147	79.07 (68.95-87.1)	45.14 (36.84-53.64)
Relative** (n=268)	103 (38.4%)	163	74.76 (65.24-82.8)	47.88 (40.05-55.78)
Without SMD (n=386)	143 (37.0%)	229	74.83 (66.89-81.7)	49.79 (43.34-56.26)
**Mozambique Total (n=218)**	84 (38.5%)	135	70.24 (59.27-79.73)	43.28 (34.76-52.11)
Household members* (n=68)	28 (41.2%)	47	78.57 (59.05-91.70)	37.50 (22.73-54.20)
Relative** (n=100)	47 (47.0%)	71	74.47 (59.65-86.06)	32.08 (19.92-46.32)
Without SMD (n=182)	69 (37.9%)	110	72.46 (60.38-82.54)	46.90 (37.45-56.52)
**South Africa Total (n=240)**	79 (32.9%)	143	80.00 (69.17-88.35)	48.45 (40.51-56.44)
Household members (n=162)	58 (35.8%)	100	79.31 (66.65-88.83)	48.08 (38.17-58.09)
Relative (n=168)	56 (33.3%)	92	75.00 (61.63-85.61)	55.36 (45.67-64.76)
Without SMD (n=204)	74 (36.3%)	119	77.03 (65.79-86.01)	52.31 (43.37-61.14)
Responded in isiXhosa (n=227)	75 (33.0%)	133	74.67 (63.30-84.01)	49.34 (41.15-57.56)

SMD=Severe Mental Disorder; CI=Confidence Intervals;

* 2 pairs (n=4 individuals) from Mozambique reported discordant information about living together and are not treated as household members;

** 5 pairs (n=10) from Mozambique reported discordant information about their relationship to one another and are not treated as relatives
